# Two-Step Laser Post-Processing for the Surface Functionalization of Additively Manufactured Ti-6Al-4V Parts

**DOI:** 10.3390/ma13214872

**Published:** 2020-10-30

**Authors:** Juliana S. Solheid, Torsten Wunsch, Vanessa Trouillet, Simone Weigel, Tim Scharnweber, Hans Jürgen Seifert, Wilhelm Pfleging

**Affiliations:** 1 Institute for Applied Materials-Applied Materials Physics, Karlsruhe Institute of Technology, 76021 Karlsruhe, Germany; hans.seifert@kit.edu (H.J.S.); wilhelm.pfleging@kit.edu (W.P.); 2 Institute for Micro Process Engineering, Karlsruhe Institute of Technology, 76021 Karlsruhe, Germany; torsten.wunsch@kit.edu; 3 Institute for Applied Materials-Energy Storage Systems, Karlsruhe Institute of Technology, 76021 Karlsruhe, Germany; vanessa.trouillet@kit.edu; 4 Karlsruhe Nano Micro Facility, H.-von-Helmholtz-Platz 1, 76344 Eggenstein-Leopoldshafen, Germany; 5 Institute for Biological Interfaces, Karlsruhe Institute of Technology, 76021 Karlsruhe, Germany; simone.weigel@kit.edu (S.W.); tim.scharnweber@kit.edu (T.S.)

**Keywords:** laser powder bed fusion (LPBF), post-processing, laser polishing, ultrafast laser, surface functionalization

## Abstract

Laser powder bed fusion (LPBF) is one of the additive manufacturing methods used to build metallic parts. To achieve the design requirements, the LPBF process chain can become long and complex. This work aimed to use different laser techniques as alternatives to traditional post-processes, in order to add value and new perspectives on applications, while also simplifying the process chain. Laser polishing (LP) with a continuous wave laser was used for improving the surface quality of the parts, and an ultrashort pulse laser was applied to functionalize it. Each technique, individually and combined, was performed following distinct stages of the process chain. In addition to removing asperities, the samples after LP had contact angles within the hydrophilic range. In contrast, all functionalized surfaces presented hydrophobicity. Oxides were predominant on these samples, while prior to the second laser processing step, the presence of TiN and TiC was also observed. The cell growth viability study indicated that any post-process applied did not negatively affect the biocompatibility of the parts. The presented approach was considered a suitable post-process option for achieving different functionalities in localized areas of the parts, for replacing certain steps of the process chain, or a combination of both.

## 1. Introduction

Over the past few years, the manufacturing industry has been directly affected by the increased environmental awareness. Aspects such as resource consumption, waste management, and pollution control have been the focus of studies concerning the environmental impact of the additive manufacturing process (AM). These investigations led to the conclusion that it is possible to create a sustainable future for the AM industry [1]. Other characteristics, i.e., reduced production lead-times and the possibility of creating complex geometries and internal features, make the AM a preferable alternative to conventional processes for specific niche areas. The mentioned benefits can be associated with the possibility for the customization and personalization of fabricated components with little impact on the cost and complexity of the manufacturing process [2]. Headed by the biomedical, aerospace, and electronic industries, AM adoption is steadily growing, especially because of the wide range of suitable materials and the design freedom offered [3]. Laser powder bed fusion (LPBF) is one AM method used for building metallic parts and it shares most of the stated advantages of AM. Although the range of materials applicable to LPBF is continually expanding, its use is limited to weldable and castable metals and alloys. The alloy Ti-6Al-4V, specifically, presents highly desired characteristics for biomedical and aerospace applications, such as a light weight, corrosion resistance, a high strength, and a high fracture toughness [4,5,6,7]. 

LPBF is often followed by post-processing stages to guarantee that the parts meet the required properties and quality. The initial high level of roughness is one of the main issues faced when adopting this type of manufacturing technique. Its origin is related to the attachment of powder particles to the surface of the workpiece and to the layer-by-layer method, which can cause a staircase effect in the vertical direction [8]. Therefore, AM components commonly endure several subsequent procedures, depending on their material composition and application. A common process chain for LPBF-built metallic components includes, not only heat treatment for microstructural modifications, but also treatments such as sandblasting, mechanical polishing, and traditional machining [9,10,11,12]. Furthermore, in the specific case of Ti-6Al-4V for medical implants, the parts can undergo superficial modifications to minimize bacterial adhesion or to increase the biocompatibility [13,14]. 

Combining AM and laser polishing is an approach that can offer a high flexibility, resulting from a variety of systems, parameters, and technologies available. In this method, the laser is focused on the workpiece and melts a thin layer of the material. The surface tension assures a uniform flow of the liquid metal, which solidifies without major irregularities, thus reducing the roughness. Together with the high throughput, the short treating time and the absence of mechanical stresses induced during the process make laser polishing a viable alternative to mechanical grinding and polishing for finishing and improving the quality of AM parts [15,16,17,18,19].

In addition to laser polishing with continuous wave (cw) lasers, the use of ultrafast lasers can also be beneficial as a finishing step for LPBF Ti-6Al-4V components. Due to the extremely high peak power, ultrafast laser sources with a pulse length shorter than 10 ps are suitable for cutting, drilling, and ablation. The exceptionally low pulse duration restricts the laser/material interaction interval, and in this way, the laser energy causes minor thermal-induced modifications in the material [20]. Parameter selection for the ablation process not only affects the material removal rate, but can also result in different surface topographies. These are commonly characterized as nano-ripples, also known as laser-induced period surface structures (LIPSS), or as porous structures generated by selective material removal. Both conditions can greatly influence the functionality of the resulting parts, by being able to modify their original wettability and/or biocompatibility [21,22,23,24,25]. 

Many efforts have been dedicated to investigating the laser functionalization of conventionally manufactured Ti-6Al-4V for bio applications. Considering the growing establishment of the AM process, some research groups are also committed to extending this approach to LPBF Ti-6Al-4V parts [26,27,28]. However, to the best of our knowledge, no work has focused on the versatile machining possibility obtained when a defined and controlled combination of different laser post-processes and parameters is used, which are capable of inducing ablation, modification by self-organized structuring, and/or re-melting, with the latter leading to planarization by material relocation.

In this context, the aim of the current research was to increase the prospect of obtaining specific surface properties by applying a two-step laser-assisted post-process, in order to precisely modify the LPBF parts made of Ti Gr 23, which is a powder with a chemical composition almost identical to Ti-6Al-4V. In particular, the approach presented here consists of analysing the resulting functionalization after laser polishing with a cw laser for roughness reduction and after structuring via a femtosecond laser procedure individually, as well as in combination. Subsequently, the samples are assessed for wettability and cell growth viability to evaluate the new surface characteristics. The outcome of this paper is a flexible and fast alternative to traditional finishing, to which the LPBF parts are usually submitted, thus adding value and new perspectives on applications, while also simplifying the process chain.

## 2. Materials and Methods

### 2.1. Material

**Figure 1:**
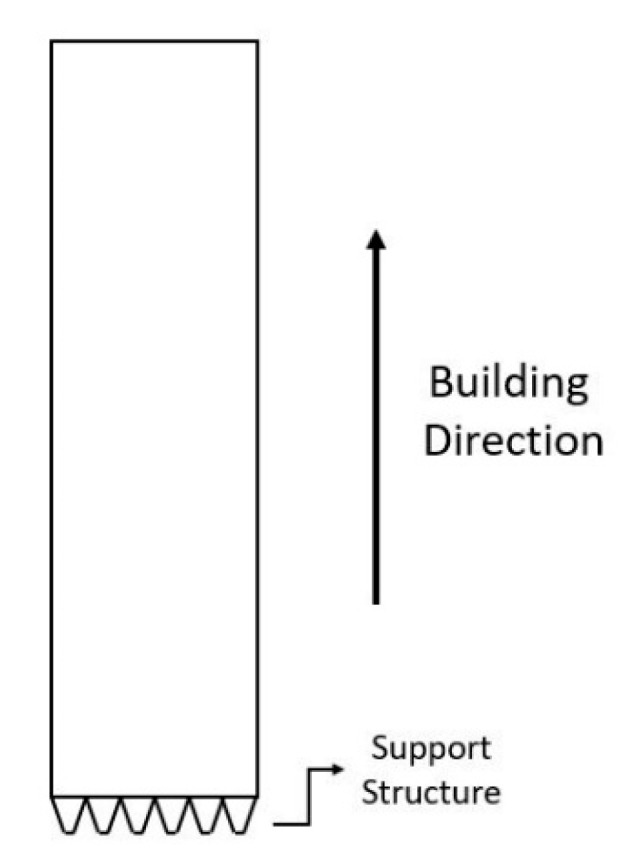
Schematic view of the laser powder bed fusion (LPBF) specimen subject to the laser post-processing steps.

The workpieces investigated in this work were produced on a DMP Flex 350 machine (3D Systems, Leuven, Belgium) using LaserForm Ti Gr23 (A). Rectangular blocks with 55 × 15 × 4 mm^3^ dimensions were manufactured in the upright position (Figure 1). The process chamber was filled with argon to provide an inert atmosphere. The manufacturer provided the processing parameters and build settings, such as the laser power, scanning speeds, and strategies. The resulting vertical surfaces, which exhibited a high degree of roughness due to powder attachment and the layer-by-layer building process, were selected for the laser post-processing investigation.

**Figure 2:**
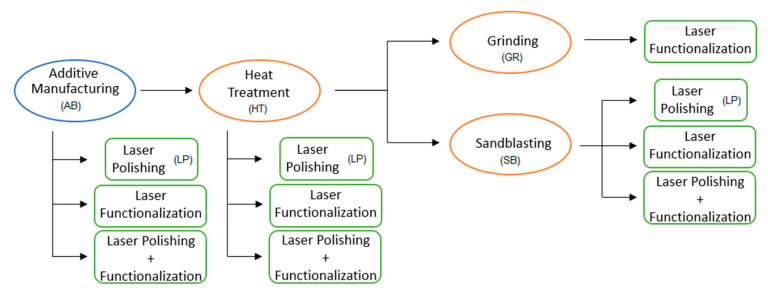
Schematic view of the different post-processing steps assessed during this work.

In this research, the topography, wettability, surface chemical composition, and cell growth viability were assessed for LPBF parts at different stages of the process chain (Figure 2). After-treatment methods commonly adopted following LPBF included heat treatment for stress relief, grinding, and sandblasting for powder particle removal. At first, samples without any previous laser treatment were submitted to such post-processes. Their starting topographies regarding the powder attachment condition were as follows: Without removal directly after additive manufacturing (AB) and heat treatment (HT); partially detached following sandblasting (SB); and completely eliminated with grinding (GR). The surfaces resulting from the LPBF and from each mentioned post-process were considered the initial conditions of the respective steps.

For a comprehensive comparison, laser polishing was performed in each of the initial conditions, with the exception of the grinding procedure. This is because, unlike other finishing methods, the grinding action is capable of reducing the roughness significantly on its own, making it unnecessary to subject the workpiece to the laser polishing routine. In contrast, every single initial condition was submitted to laser functionalization. By employing this process, two types of topographies were obtained: LIPSS and pores. The novel proposed combination of laser polishing and laser functionalization, as the LP, was not applied to the ground surface.

### 2.2. Laser Systems

#### 2.2.1. Continuous Wave Laser

The laser polishing task was carried out by a TruCell 3010 machine integrated with a radiation source (TruDisk 3001, TRUMPF GmbH, Ditzingen, Germany), which uses a laser wavelength of 1064 nm and operates in a continuous wave (cw) mode. The lenses (BEO D70) work with a focal length of 150 mm and focus the laser beam towards the top facet of the part. In the course of the procedure, the material is prevented from oxidizing by a single nozzle responsible for blowing a flow of argon directly on the sample’s surface. The parameter set, utilized in this work, was selected based on previous process optimization and material characterization studies for resulting properties. Table 1 introduces the adopted parameters [29,30].

#### 2.2.2. Ultrafast Pulsed Laser

The structuring was performed in an ultrafast fiber laser system (Tangerine, Amplitude Systèmes, Bordeaux, France), with a near infrared wavelength of 1030 nm and without shielding gas. The primary step of this research was to use the different sets of ultrashort laser parameters to create two types of structures: Pores and nano-ripples. The values used to achieve both desired structures are presented in Table 2.

To minimize the material ablation and, consequently, maintain the samples within the geometrical tolerances that are often required in manufacturing environments, a low laser power is vital. The higher energy input required to obtain the porous structure was thus obtained by increasing the number of repetitions from 1 to 5.

### 2.3. Wettability

An automatic, video-based, contact angle analysis system (Dataphysics, OCA 40Mirco, Filderstadt, Germany) was used to analyse the wetting behavior. For each measurement, distilled water drops with a volume of 1 μL were dispensed with a dosing rate of 0.7 µL/s. The evolution of the contact angles was recorded in a video and the quantitative analysis was obtained within 10 s of the droplet/surface contact.

### 2.4. X-ray Photoelectron Spectroscopy

For the surface chemical composition analysis, X-ray photoelectron spectroscopy (XPS) measurements were performed using a K-Alpha+ XPS spectrometer (ThermoFisher Scientific, East Grinstead, UK). The Thermo Avantage software was used for data acquisition and processing. All samples were analyzed using a microfocused, monochromated Al Kα X-ray source (400 µm spot size). The K-Alpha+ charge compensation system was employed during analysis, using electrons of 8 eV energy and low-energy argon ions to prevent any localized charge build-up. The spectra were fitted with one or more Voigt profiles (BE uncertainty: ±0.2 eV) and Scofield sensitivity factors were applied for quantification [31]. All spectra were referenced to the C 1s peak (C–C, C–H) at a 285.0 eV binding energy controlled by means of the well-known photoelectron peaks of metallic Cu, Ag, and Au, respectively.

### 2.5. Biocompatibility

The influence of the different post-processes on the part’s biocompatibility was assessed by analysing the viability of MC3T3-E1 cells (DSMZ ACC 210, Braunschweig, Germany). This murine pre-osteoblast cell line is widely used as a model system in bone research. As implants for hard tissue represent a potential application for the Ti-6Al-4V parts, this cell line was chosen for the biocompatibility tests in this study. MC3T3-E1 cells were cultivated in alpha-minimal essential medium (MEM) supplemented with ribonucleosides, deoxyribonucleosides, 2 mM L-glutamine, 1 mM pyruvate, 10% (v/v) fetal calf serum, and 1% penicillin/streptomycin (all Gibco, Invitrogen, Karlsruhe, Germany) in an atmosphere of 5% CO_2_. 

In order to find a cytotoxic effect, an MTT assay was performed. The assay uses the ability of metabolically active cells to reduce the water-soluble tetrazolium salt MTT (3-(4,5-dimethylthiazol-2-yl)-2,5-diphenyltetrazolium bromide, Sigma-Aldrich, Taufkirchen, Germany) to a purple colored formazan. The intensity of the dye is proportional to the number of metabolically active cells. For this assay, MC3T3-E1 cells growing in a microwell plate were exposed to extracts of the samples. In the case of the release of toxic substances from the samples, the cells would be harmed and their metabolic activity reduced. 

In this study, 7.5 × 10^3^ MC3T3-E1 were seeded into each well of a 96-well plate and incubated for 24 h. The samples were extracted by immersing them in complete cell culture medium for 24 h under cell culture conditions (37 °C, 5% CO_2_, approx. 100% humidity). The extracts were tested in two concentrations: Pure (100%) and diluted (10%, the extracts were diluted 1:10 with fresh cell culture medium). As a negative control, cell culture medium was used and treated as the samples (24 h cell culture conditions). Additionally, as a positive control, copper discs were used and extracted as the samples. In all cases, the cell culture medium was carefully removed and replaced by 100 µL of the extracts or controls. All samples were tested in quadruplicate. The cells were incubated for another 24 h. Afterwards, 50 µL of the MTT solution (1 mg/mL in MEM) was added and incubated for 4 h. Finally, the cell culture medium was removed and the water-insoluble formazan dissolved by adding 100 µL 2-propanol. The absorbance was measured using a spectro-photometer (Synergy H1, BioTek, Winooski, VT, USA) at 570 nm, with a reference wavelength at 650 nm. 

In a second series of tests, MC3T3-E1 cells were cultured on top of the parts and their viability and morphology were assessed with a fluorescence microscope. For cleaning and sterilization, the laser textured samples were sonicated in ethanol in an ultrasound bath and afterwards, were placed inside a 24-well culture plate. Cells were seeded onto the samples by adding 1.5 mL of cell suspension with a cell concentration of 6.5 × 10^6^/mL into the well. After incubation for 24 h, cell culture medium was removed, and the cells were washed with PBS and fixed by incubating the cells for 30 min with 2% glutaraldehyde (Sigma-Aldrich, Taufkirchen, Germany) in PBS. Fixation with glutaralehyde induces autofluorescence in cells, allowing observation of the cells under a fluorescence microscope, without further staining [32]. Additionally, the cell nuclei were stained using Hoechst 33342 (Invitrogen, Karlsruhe, Germany). Cell images were obtained using a fluorescence microscope (Axiovert 200M, Zeiss, Oberkochen, Germany). 

## 3. Results

### 3.1. Surface Characterization by Scanning Electron Microscopy

**Figure 3:**
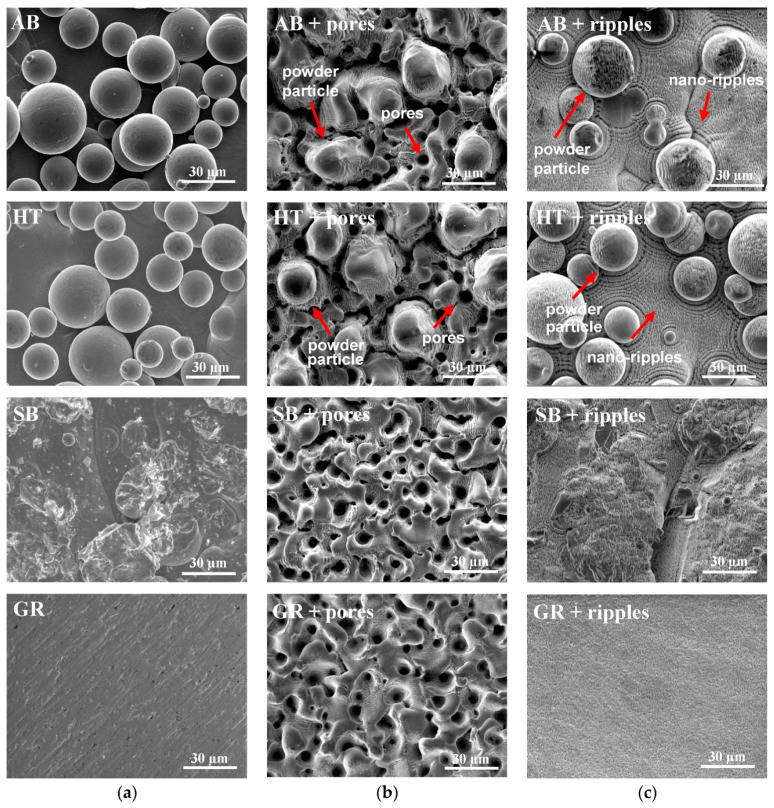
SEM images of the surfaces: (**a**) Without laser treatment; (**b**) with ultrafast laser treatment for pore formation; and (**c**) with ultrafast laser treatment for nano-ripple formation.

It is expected that the adoption of diverse laser systems, parameters, and a combination of varied processes results in distinct topographies. The initial topography greatly influenced the laser/material interaction, and by using the exact same parameters for the samples with varied starting conditions, different results were obtained (Figure 3).

The ultrashort laser alone was not capable of removing the irregularities on the samples. Instead, when powder remained sintered, i.e., AB and HT starting conditions, the nano-ripples were formed both in the part’s and particle’s surfaces (Figure 3c, AB + ripples and HT + ripples), while residues of such adjunct were still detected among the porous structure formation (Figure 3b, AB + pores and HT + pores). The generation of structures that are more homogeneous is possible when the presence of sintered particles on the surface is partially or completely eliminated in advance (Figure 3c, SB + ripples and GR + ripples). The pores formed are very similar in size and distribution when sandblasting and grinding are the preceding procedures (Figure 3b, SB + pores and GR + pores), while for the nano-ripple formation, the fact that the initial topography could not be completely flattened through sandblasting is still evident (Figure 3c, SB + ripples).

The following step of this research was to submit the parts with the starting conditions described previously to laser polishing (except for the sample smoothened via grinding), and combine it with the ultrafast structuring process.

**Figure 4:**
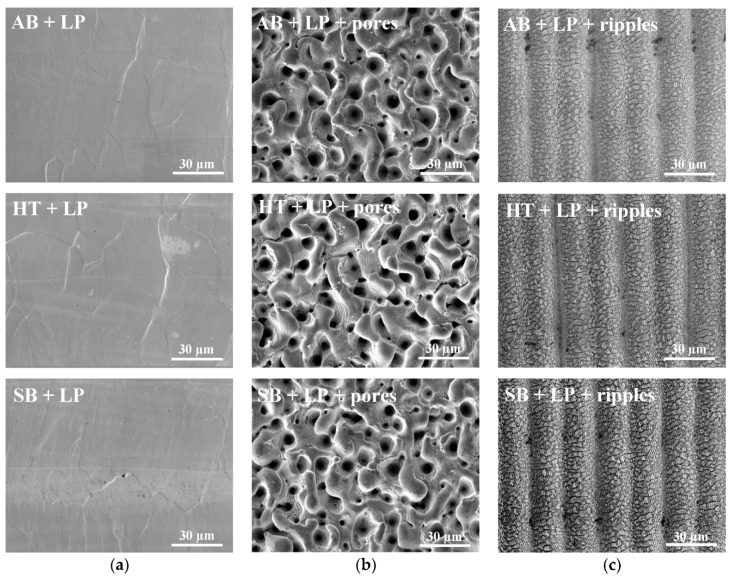
SEM images of the surfaces: (**a**) After laser polishing; (**b**) with ultrafast laser treatment for pore formation; and (**c**) with ultrafast laser treatment for nano-ripple formation.

As demonstrated in previous works [15,16,17,18,19], laser polishing successfully removed the particles from the part’s surfaces and flattened the initial topographies, giving very consistent results for all cases. Similarly, the structuring results were regular and evaluated as having a high repeatability (Figure 4). The laser polished surfaces are also considered starting conditions when assessing the functionalization step.

For the porous structure, the results resembled the samples finished by sandblasting and grinding, which indicates a high stability and repeatability for laser functionalization. The nano-ripples were formed similarly on all laser polished workpieces, although the configuration is clearly different from the, also smooth, ground part. Even with the same parameters, when subjecting the material to nano-ripple formation, following the laser polishing, there was evidence for the beginning of overheating. This fact indicates that not only the initial topographies, but also the different processes to which they were submitted, impact the laser/material interaction and can alter their characteristics, such as the reflectivity and absorptivity of the laser radiation, affecting the results.

**Figure 5:**
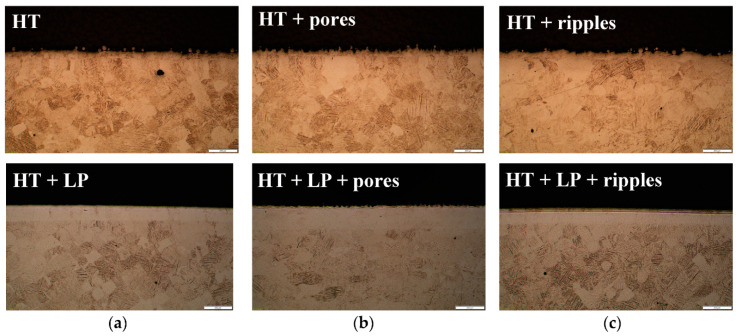
Cross-section images of the heat-treated samples: (**a**) Before and after laser polishing; (**b**) with ultrafast laser treatment for pore formation; and (**c**) with ultrafast laser treatment for nano-ripple formation.

The difference in the topographies can also be observed in cross-sectional images (Figure 5). The heat-treated samples subjected to the laser post-processes, combined and separated, were selected to illustrate the varied topographies obtained and the potential microstructural changes caused during each process. The laser polishing removed the attached powder, while causing the occurrence of heat-affected zones (Figure 5a). The pore formation resulted in irregular surfaces for the rough or smoothened samples and caused no change in the microstructure (Figure 5b). Finally, when nano-ripples were generated, there were no alterations in either the microstructure or the topography condition, since the rough and smoothed surfaces remained in the same state (Figure 5c). The same behavior could be observed for every sample that was previously submitted to heat treatment, while no microstructure modifications were observed in the AB samples after any laser post-processing [30].

### 3.2. Wettability

**Figure 6:**
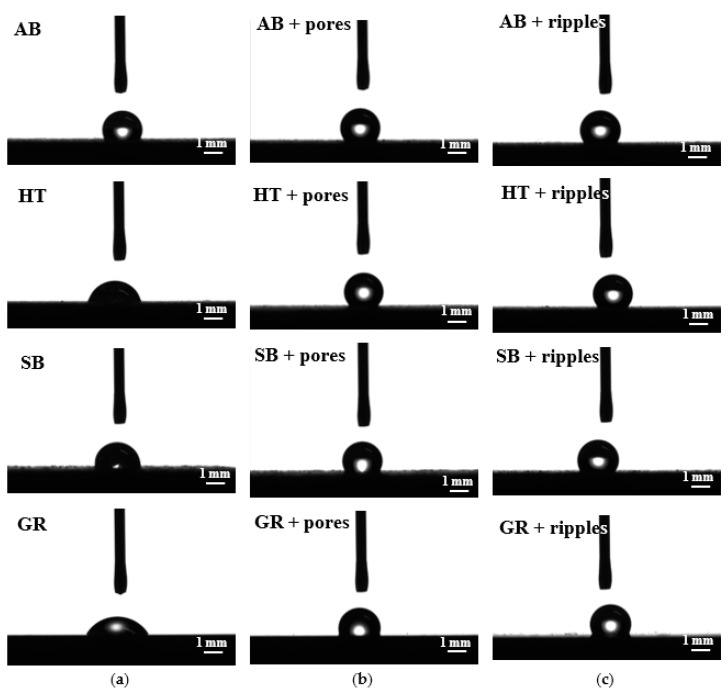
Water drop images from wettability analysis of the surfaces: (**a**) Without laser treatment; (**b**) with ultrafast laser treatment for pore formation; and (**c**) with ultrafast laser treatment for nano-ripple formation.

**Figure 7:**
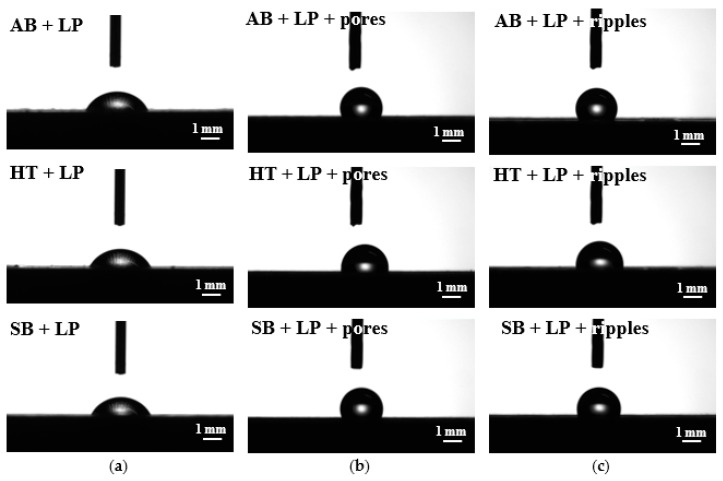
Water drop images from wettability analysis of the surfaces: (**a**) After laser polishing; (**b**) with ultrafast laser treatment for pore formation; and (**c**) with ultrafast laser treatment for nano-ripple formation.

In terms of specific applications, the impact of the manufacturing, or post-processes, on the functionality of the parts is a major concern. For this reason, the investigated samples were assessed for their wettability characteristics, subsequent to all of the stages described in the previous section. In this case, the different topographies obtained with and without any laser post-treatment resulted in different wetting behaviors (Figure 6 and Figure 7). 

**Figure 8:**
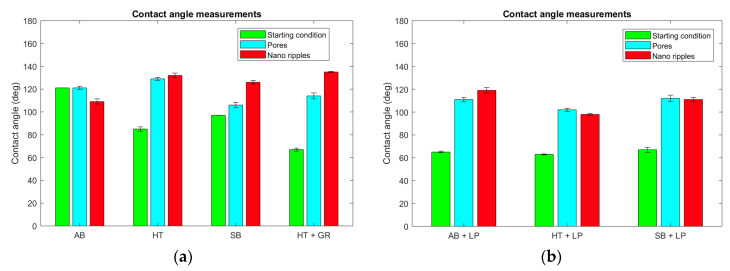
Contact angle comparison between ultrafast laser treated AM Ti Gr 23 samples and their initial conditions: (**a**) Individually; and (**b**) combined with laser polishing.

The evaluation performed throughout this work was conducted after AM and prior to laser post-processing in four initial conditions. Despite their similar topographies, the as-built and heat-treated workpieces showed fairly distinct wettability results, with an average contact angle (CA) of 121° and 85°, respectively (Figure 8a). When the traditional powder removal techniques were applied, different performances were observed, with a CA of 97° for the sandblasted and 67° for the ground samples. 

After the ultrafast laser treatment, the surfaces presented hydrophobic characteristics, independent of the initial or resulting topographies. The CA values were always above 100°, reaching up to 135° for the ground heat-treated sample with nano-ripple formation. Although the hydrophobicity is a common factor of both types of structures formed with ultrashort laser processing, the occurrence of nano-ripples induced a general tendency of obtaining a higher CA when compared to the porous structures, with the exception of the as-built condition (Figure 8a). When compared to previous studies focused on the laser functionalization of conventionally manufactured Ti-6Al-4V [21,23], the hydrophobicity of the parts subjected to ultrashort pulsed laser presents the same behavior.

Laser polishing was the third technique adopted for powder removal and surface quality improvement. As previously mentioned, the resultant surfaces after laser polishing were very much alike, even though the starting conditions differed. The same could be observed for the wettability results (Figure 7). As-built, heat-treated, and sandblasted samples, subjected to laser polishing, obtained CA values of around 65° (Figure 8b). The combination of laser polishing and ultrashort processing resulted in hydrophobic characteristics for both nano-ripple and porous formation. Similar to the ultrashort laser-treated parts without laser polishing, the CA always presented values above 100°, with a maximum value of 121° for the as-built workpiece submitted to laser polishing and with nano-ripple formation. In contrast to what was discussed earlier, no significant difference could be observed in the CA measurement values for the surfaces with the occurrence of nano-ripples or pores.

### 3.3. Surface Chemistry Analysis

**Figure 9:**
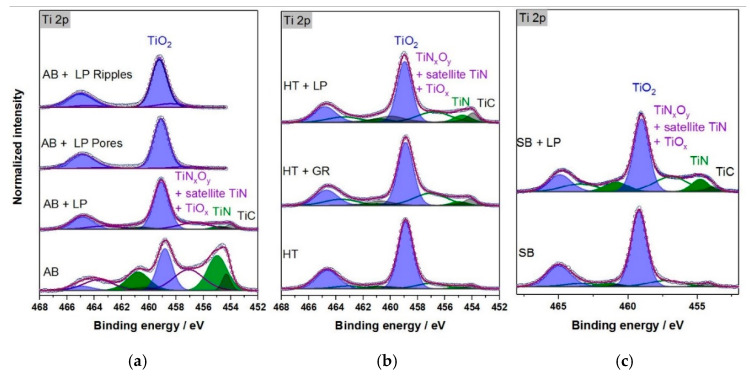
Ti 2p XPS spectra for different processing steps with the following starting conditions: (**a**) Additive manufacturing; (**b**) heat treatment; and (**c**) sandblasting.

The wetting behavior of the parts cannot be simply related to the topography. For that reason, the surface chemistry following each process discussed was obtained through XPS analysis. All parts were kept under ambient air for several days after their respective procedures and, as expected, the passivation commonly observed in titanium once in contact with air can be shown by the presence of doublets attributed to TiO_2_ (Figure 9, Ti 2p_3/2_ at 459.0 eV) and TiO_x_ (Figure 9, ~456.5 eV) [33]. Although not illustrated, other metal oxides, such as Al_2_O_3_ and V_2_O_5_, were also detected on the surfaces of varied workpieces. The combination of metal oxides, in different concentrations, formed a layer in every sample, despite the process to which it was submitted. 

Amongst the conditions prior to any laser treatment and after laser polishing, further doublets were detected, with Ti 2p_3/2_ at 454.2, 455.0, and 457.0 eV corresponding to TiC, TiN with its satellite [34], and TiN_x_O_y_, respectively. The matching C 1s peak of TiC could be found at 282.3 eV, whereas N 1s of TiN appeared at 396.4 eV (not shown here). The highest amount of TiN and TiC detected occurred after additive manufacturing, which took place under an inert atmosphere provided by an argon filled chamber. This was unlikely to have been caused during the LPBF process, and the discrepant nitrogen content on the AB parts could be the result of an external interaction with titanium after the process. Subsequent procedures, such as heat treatment and sandblasting, led to less noticeable peaks of TiN and TiC. The same is true for the ground samples, although in this case, the TiN and TiC peaks were more pronounced when compared to the heat-treated and sandblasted samples. All three steps were performed without inert atmospheres.

As described in Section 2.2.1, during the laser polishing, a stream of argon was blown directly onto the workpiece to avoid oxidation. The TiN and TiC, in these cases, had a higher concentration when compared to the heat-treated, sandblasted, and ground parts. One possible explanation for this could be the fact that, once the laser polishing process is over, the argon flow stops, yet the material remains at extremely high temperatures for several minutes, reacting with the air in its surroundings. Nevertheless, these contents were still lower when compared to the samples after additive manufacturing in inert atmosphere.

The surface chemical analysis following the ultrafast laser processing are represented by the peak detection for ripple and pore formation displayed in Figure 9a. In all functionalization cases, mainly metal oxides were detected on the surfaces, whereas TiC and TiN were no longer present. These findings could be expected as the process was performed without shielding gas or inert atmospheres. Despite being considered a “cold ablation” approach, for a few tenths of seconds, the temperatures can reach thousands of degrees within a nm-scale surface layer, different from laser polishing. Furthermore, as a consequence of the material removal, the appearance of vanadium oxide peaks could be observed for all functionalized surfaces, with a main component at 517.2 eV (V_2_O_5_), accompanied by a weaker component at 515.2 eV (probably V_2_O_3_) [35]. 

The surface chemical composition acquired after each step of the process chain is summarized in Table 3. It is important to take into consideration that the XPS analysis is limited to a few nanometers in depth and the element concentrations obtained are thus not related to the bulk material. Part of the oxygen, carbon, and nitrogen content detected emerged from surface contamination (cont.) [36], while the remaining content developed due to a reaction with the core elements.

Even when comparing the concentration of titanium, aluminum, and vanadium combined, oxygen and carbon were the elements with higher concentrations for all cases. Although its presence was unexpected, the total amount of nitrogen on the surface of the AB parts before and AB, SB, and HT parts after laser polishing was relatively high, with values between 6 and 8 at%, mainly due to the presence of TiN and TiN_x_O_y_. After functionalization, almost no nitride and oxynitride were detected and the relatively low nitrogen content was only due to contamination (1 to 2 at%). Likewise, most of the oxygen content stemmed from oxidation of the metals (O 1s at 530.4 eV).

**Figure 10:**
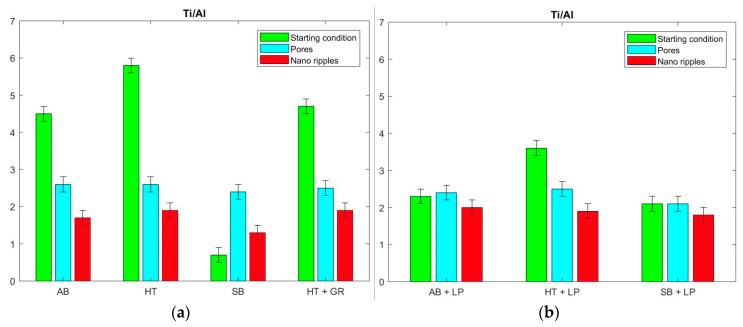
Ti:Al ratio comparison between ultrafast laser treated AM Ti Gr 23 samples and their initial conditions: (**a**) Individually; and (**b**) combined with laser polishing.

In order to consider the material composition itself, it is important to disregard the contamination through a quotient analysis of the basic components of the metal alloy, which allows the element content behavior on the parts’ surface to be understood for each manufacturing step adopted. The Ti:Al ratios for the AB, HT, SB, and GR samples were quite distinct, with values of 4.5, 5.8, 0.7, and 4.7, respectively (Figure 10a). The significant alteration in the elemental composition revealed a rise in the aluminum concentration after the sandblasting. However, the amount of titanium and aluminum remained in the same range for the further processes.

When submitted to laser polishing, the AB, HT, and SB workpieces exhibited a Ti:Al proportion of 2.3, 3.6, and 2.1, respectively (Figure 10b). The most relevant change occurred on the SB parts, in which a substantial growth of titanium, alongside a reduction of aluminum, led to a significantly higher quotient. In contrast, a notable increase of aluminum contributed to lower ratios on AB and HT samples. A similar behavior was observed for the surfaces with pore and nano-ripple formation for all cases, resulting in a Ti:Al proportion of around 2.2 ± 0.2 (Figure 10a,b). In the same way, the ratio Ti:V can be assessed. While in the first processing steps, no vanadium oxide could be detected in most cases (Table 3), all pore and nano-ripple functionalized workpieces showed a similar Ti:V ratio of 20 ± 2, which is comparable to the theoretical value of 24 calculated from the expected stoichiometry. Therefore, the final outcomes, namely Ti:Al:V proportions, were comparable in every condition, regardless of the type of laser process to which they were subjected.

### 3.4. Biocompatibility

Since titanium and its alloys are often adopted for the manufacturing of biomedical devices because of their superior biocompatibility and mechanical properties, it is important to guarantee that laser post-processing for surface quality improvement, functionalization, or a combination of both does not decrease the biocompatibility of the material.

**Figure 11:**
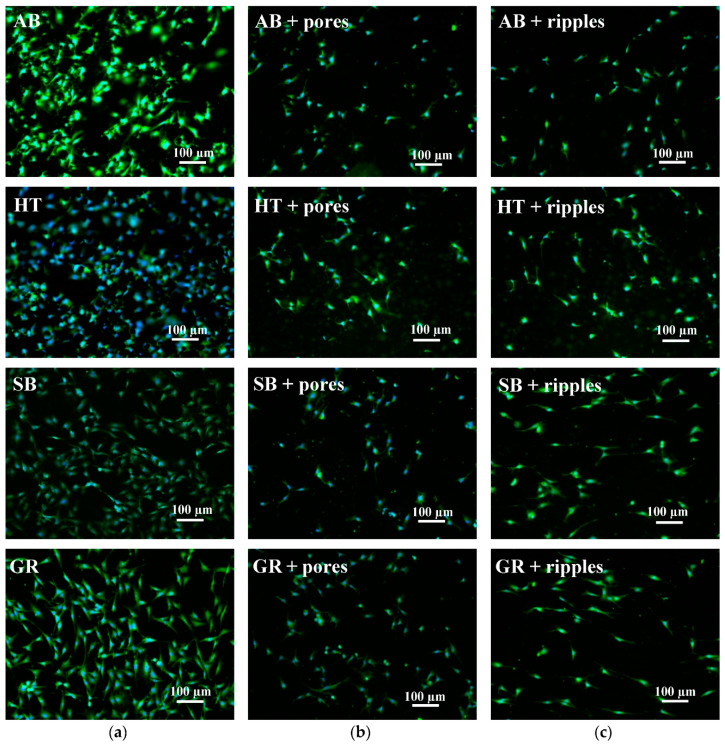
Fluorescence images of MC3T3-E1 cells growing on the surfaces of parts: (**a**) Without laser treatment; (**b**) with ultrafast laser treatment for pore formation; and (**c**) with ultrafast laser treatment for nano-ripple formation.

**Figure 12:**
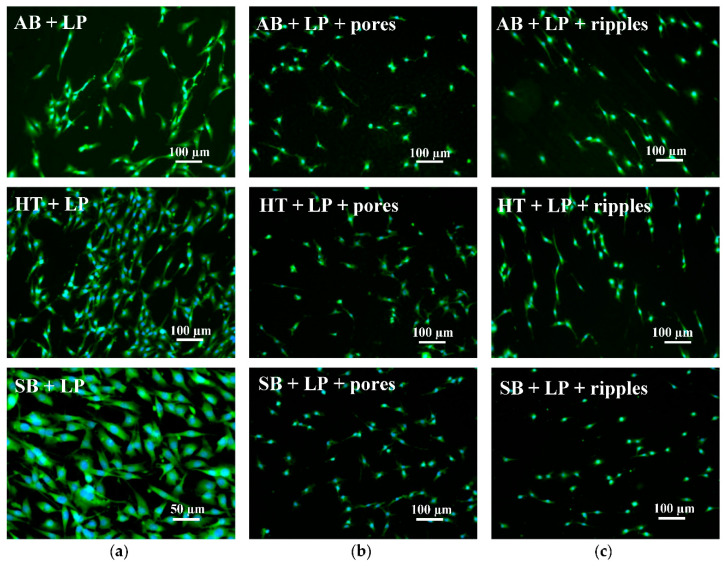
Fluorescence images of MC3T3-E1 cells growing on the surfaces of parts: (**a**) Without laser treatment; (**b**) with ultrafast laser treatment for pore formation; and (**c**) with ultrafast laser treatment for nano-ripple formation.

The fluorescence images from cells grown on the parts (Figure 11 and Figure 12) showed that laser functionalization neither prior to nor after laser polishing had adverse effects on the capability of cells to attach and grow on the part’s surface. In all cases, the cells also displayed a normal morphology.

In all cases displayed in Figure 11, the orientation of attached cells seems arbitrary. The same can be observed for the laser polished surfaces in Figure 12a,b, prior to and after porous structure formation. However, when nano-ripples were obtained after the two-step laser post-process, the cells showed a slight tendency to grow in a specific direction (Figure 12c), which coincided with depression lines due to the starting of overheating.

**Figure 13:**
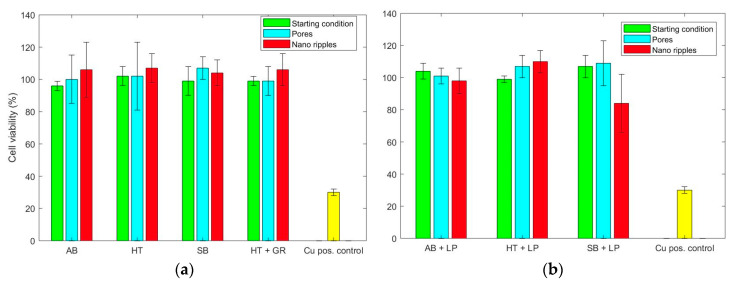
Cell viability (in percentage) of MC3T3-E1 cells cultivated with the extracts from ultrafast laser treated AM Ti Gr 23 samples compared with the negative control (100% viability) and their initial conditions: (**a**) Individually; and (**b**) combined with laser polishing.

The results of the toxicity analysis obtained with an MTT assay are summarized in Figure 13. The viability of the cells on the samples is displayed as the percentage of negative controls. In most cases, the cells being cultivated with the extracts displayed the same viability as the controls, which implies that the investigated parts retained their high biocompatibility, even after the different post-processes. Similar results were obtained after the laser functionalization of conventionally manufactured Ti-6Al-4V [22,23].

## 4. Discussion

To achieve the design requirements, the LPBF process chain can become long and complex. Research has been performed to identify bottlenecks and improve the efficiency of this type of manufacturing as a whole [37]. While several techniques are under investigation for increasing the productivity and precision of the LPBF process [38,39,40], little attention has been paid to solving issues in the subsequent procedures.

The present study focused on the modification of the surface topography and functionality of samples submitted to two-step laser post-processing, following different established steps of the LPBF process chain.

As mentioned, the use of ultrafast laser processing to modify the wettability or biocompatibility of traditionally manufactured Ti-6Al-4V has been explored in many studies [21,22,23,24,25]. When such parts are produced via LPBF, different considerations must be taken into account, e.g., at which stage to employ the laser functionalization. The heat treatment applied for stress relief is an essential stage, as it results in powder removal for surface quality improvement. Laser polishing has been proven to be a technique able to achieve the latter [15,16,17,18,19], while affecting the former [30]. A combination of laser polishing and laser functionalization, and an assessment of their effect throughout the LPBF process chain, has not yet been evaluated in detail.

The samples, prior to any laser finishing, could be assessed by the stage to which they belong. After the LPBF, there was excessive attachment of powder to the part’s surface, which resulted in a high roughness. The mentioned workpiece presented the highest amount of TiN and TiC, in addition to the metal oxides, despite the controlled inert atmosphere adopted during the process. As stated, this is likely due to external reactions following the LPBF. At this step, the surface was hydrophobic. In contrast, the heat-treated parts were more hydrophilic, yet the AB and HT topographies were almost identical. The TiN content was a lot lower after heat treatment, in comparison with the as-built one. Since the workpieces came from the same batch, it is believed that this divergence was due to the temperatures applied during the stress relief process as these were above the temperature employed for the decomposition of TiN [41].

The sandblasting and grinding steps were performed following the heat treatment and, even though no inert atmosphere was used, the relatively low temperatures involved in the processes prevented any further reactions of the titanium alloy with nitrogen. Therefore, there were similar contents of TiN, TiC, and metal oxides after these stages in comparison with the heat-treated sample. The comparable wetting behavior of the three parts reinforces the concept of chemical composition having a great impact on the surface wettability. 

As already described in previous research, the adoption of ultrashort pulse lasers with the goal of achieving nano-ripples or porous structures can, respectively, have almost no effect on the surface texture or increase it drastically, depending on the starting conditions [42]. The same was observed in this study, where rough parts (AB and HT) maintained high asperities for both created structures, while smoothened faces (SB, GR, and all LP) acquired a high texture when pores were obtained. The nano-ripples did not affect the texture of the flattened samples on a micro scale.

With nano-ripple formations, different topographies were acquired, although the wetting behaviors were comparable, as were the chemical compositions of the modified surfaces. The high temperatures involved in the process and the lack of an inert atmosphere led to the removal of TiC and TiN and to the formation of metal oxides (Ti, Al, and V), which, combined with the topographies, determined the hydrophobic performance, in these cases [43]. Since porous surface creation occurred in the same conditions as for the nano-ripples, the chemical composition was also similar, as were the equivalent wettability characteristics. Nevertheless, the topographies were very distinct. 

Using the laser polishing as a step prior to functionalization led to highly repeatable and controllable results, since the process was capable of bringing samples with completely different conditions in terms of the topography and surface chemical composition to the same initial state. In contrast to the ultrafast processing, the laser polishing caused not only the expected metal oxides, but also TiC and TiN.

After comparing all presented conditions, the wettability/chemical composition/topography dependency can be better elucidated. In general, flat samples with the presence of metal oxides in their surfaces tend to be hydrophilic, while rough parts (even on a nano scale) with a similar superficial chemical state have a tendency to be hydrophobic [43], which was the exact behavior observed during the development of this research for the samples submitted to both laser post-processes, separately or combined.

Patterned topographies can play an important role as scaffolds and aid in cell proliferation [44]. This type of performance was not observed for the structures obtained during this study; although the cell growth viability indicates that any post-process applied does not negatively affect the biocompatibility samples. With this feature unaffected, laser polishing and functionalization can be either combined or used independently, in order to replace certain steps of the process chain and to achieve different functionalities, such as hydrophilicity and hydrophobicity, in localized areas of the parts.

## 5. Conclusions

The combination of laser polishing and ultrafast laser functionalization has not been previously explored for titanium alloys specifically built via LPBF. The presented approach is considered a suitable post-process option for achieving different functionalities in localized areas of the LPBF parts, for replacing certain steps of the process chain, or a combination of both. Our results demonstrate that laser polishing is capable of bringing samples from distinct topographies to the same initial state, after which the structures obtained by laser functionalization can be better controlled and more repeatable. The same is true for the wettability behavior of the workpieces, with the resulting laser polished surfaces presenting contact angles in equivalent ranges before (hydrophilic) and after (hydrophobic) modification with the ultrafast pulsed laser. Based on the XPS analysis, such similarity in the wettability is related to the comparable surface chemical composition obtained after each laser post-process studied, and not only to the topographies obtained. In all cases, the biocompatibility is not negatively affected. Further examinations are needed to assess the adhesion of different types of cells, based on the regular bio application of Ti-6Al-4V LPBF devices. 

## Figures and Tables

**Table 1:** Process parameters employed for laser polishing.

Parameter	Value
Laser wavelength	1064 nm
Laser power	300 W
Focal length	150 mm
Focal position	+1 mm
Scanning speed	800 mm/s
Axial feed rate	0.3 m/min
No. of repetitions	5

**Table 2:** Process parameters for surface structuring.

Parameter	Value for Pore Structures	Value for Nano Ripples Structures
Laser wavelength	1030 nm	1030 nm
Average laser power	4 W	4 W
Scanning speed	400 mm/s	400 mm/s
Repetition rate	1000 kHz	1000 kHz
Pulse duration	450 fs	450 fs
Focal length	100 mm	100 mm
No. of repetitions	5	0

**Table 3:** Main chemical components detected on the surface of different samples (at.%).

Sample	Ti 2p	Al 2p	V 2p	O 1s (Metal Oxide)	N 1s (TiN, TiN_x_O_y_)	O 1s Cont.	C 1s	N 1s Cont.
AB	14.1	2.9	-	10.8	5.8	18.5	40.2	2.1
AB + LP	11.1	4.8	-	12.2	5.3	18.0	43.2	2.3
AB + LP + pores	10.9	0.8	0.6	26.3	0.2	13.9	39.6	1.2
AB + LP + ripples	9.8	4.8	0.5	23.4	-	15.4	43.0	1.2
HT	13.5	2.3	0.8	27.4	1.3	14.6	36.6	1.0
HT + GR	15.1	3.2	0.7	25.4	3.6	14.3	34.6	1.5
HT + LP	16.3	4.8	0.3	28.4	3.3	12.4	31.3	1.5
HT + LP + pores	11.3	4.6	0.6	26.1	0.1	14.4	39.0	1.2
HT + LP + ripples	10.5	5.4	0.6	25.1	0.2	15.3	40.1	1.0
SB	6.9	10.0	-	18.9	0.5	21.0	38.0	0.9
SB + LP	13.6	6.6	0.1	24.5	4.2	14.0	31.9	1.6
SB + LP + pores	8.8	6.6	0.2	21.2	0.1	17.6	40.3	1.2
SB + LP + ripples	6.0	3.3	0.3	14.2	-	18.5	51.8	1.6
